# Novel Synthesis Approach for Natural Tea Polyphenol-Integrated Hydroxyapatite

**DOI:** 10.3390/ph17020251

**Published:** 2024-02-15

**Authors:** Xiaoxiang Ren, Zeng Yi, Xudong Li

**Affiliations:** 1 Institute of Translational Medicine, Shanghai University, Shanghai 200444, China; 2 National Engineering Research Center for Biomaterials, Sichuan University, Chengdu 610064, China

**Keywords:** tea polyphenol, hydroxyapatite, nanomaterials, mineralization

## Abstract

Hydroxyapatite (HAP) has garnered considerable interest in biomedical engineering for its diverse applications. Yet, the synthesis of HAP integrated with functional natural organic components remains an area ripe for exploration. This study innovatively utilizes the versatile properties of tea polyphenol (TP) to synthesize HAP nanomaterials with superior crystallinity and distinct morphologies, notably rod-like structures, via a chemical deposition process in a nitrogen atmosphere. This method ensures an enhanced integration of TP, as confirmed by thermogravimetric (TGA) analysis and a variety of microscopy techniques, which also reveal the dependence of TP content and crystallinity on the synthesis method employed. The research significantly impacts the field by demonstrating how synthesis conditions can alter material properties. It leads the way in employing TP-modified nano-HAP particles for biomedical applications. The findings of this study are crucial as they open avenues for the future development of tailored HAP nanomaterials, aiming at specific medical applications and advancements in nanotechnology.

## 1. Introduction

Hydroxyapatite (HAP), a bioceramic that echoes the composition of natural bone, stands at the forefront of biomaterials science, commanding the attention of researchers and clinicians alike [1,2,3]. Its inherent biocompatibility is a cornerstone characteristic, making it a favored choice for a multitude of biomedical applications, including bone grafts, dental implants, and as a scaffold for tissue engineering [4]. The osteoconductive nature of HAP, which actively promotes bone growth by mimicking the host’s natural bone mineral, is pivotal in its widespread use in orthopedics, where it assists in the healing and integration of implants with the native bone [5,6]. In dentistry, HAP’s structural resemblance to tooth enamel and bone has led to its incorporation in a variety of restorative and regenerative practices, enhancing the longevity and functionality of dental work [7,8]. Additionally, the role of HAP in drug delivery systems cannot be overstated, where its compatibility with human biology allows for targeted delivery and controlled release of therapeutic agents, thus optimizing treatment efficacy and minimizing side effects [9,10,11].

The advent of nanotechnology has bestowed HAP with new dimensions of utility, particularly when this mineral is manipulated at the nanoscale and combined with bioactive molecules [12]. This nanoscale manipulation has unlocked transformative potentials, such as the creation of more reactive surfaces for protein adhesion, leading to faster tissue regeneration and integration [13]. Tea polyphenol (TP), a constituent richly found in green tea, has garnered acclaim as a potent natural antioxidant and stands prominently in the current surge of biomaterial advancements [14,15,16]. TP encompasses a variety of polyphenolic compounds such as catechins, flavonoids, tannins, and theaflavins, each possessing multiple phenolic rings and hydroxyl groups [17,18,19]. These structural moieties are adept at donating electrons, which allows TP to effectively scavenge and neutralize free radicals, the reactive molecules that can cause cellular damage and contribute to chronic diseases [20,21]. Recognized for its role in augmenting the biological functions of the materials it is combined with, TP is poised to imbue HAP with a suite of therapeutic capabilities. When HAP is interfaced with TP, the resultant nanocomposites are expected to exhibit not only increased osteogenic activity, crucial for bone formation and healing, but also potent anti-tumor properties. The anticipation surrounding these TP-enhanced HAP nanocomposites is well-founded, as they propose to elevate the realm of biomaterials, merging the structural benefits of HAP with the biological benefits of TP to craft a new class of multifunctional, bioactive materials [22,23].

The burgeoning field of tissue engineering constantly seeks to harness the benefits of combining natural biomolecules with synthetic materials to create more biologically active and mechanically robust materials for bone regeneration [24]. For instance, polyphenols have been applied as a coating on the surface of hydroxyapatite, demonstrating anti-inflammatory capabilities [25,26]. TPs are distinguished by their antioxidant, anti-inflammatory, and anti-tumor properties, potentially reducing oxidative stress and inflammation at the site of injury, thereby creating an optimal environment for bone healing. The structural and mechanical properties of HAP, critical determinants of its efficacy as a bone substitute, are also expected to be positively influenced by the incorporation of TP [27]. This modification may alter the material’s porosity, crystallinity, and mechanical strength, attributes that significantly impact its ability to integrate with existing bone and withstand physiological stresses [28]. This introduction aims to highlight the innovative strategy of augmenting HAP with TP to create a composite material that combines the osteoconductivity of HAP with the biological advantages of TP [29]. A novel synthesis approach in this domain involves the modification of HAP with TP, which appears to be a particularly promising avenue. The study introduces a groundbreaking synthesis method for HAP by incorporating TP, utilizing chemical precipitation to precisely tailor nanoparticle attributes, such as size, shape, and crystalline structure. This innovative approach aims to produce TP-enriched HAP nanocomposites, exploring the impact of synthesis conditions on their properties. The expected synergy between HAP and TP has the potential to improve the bioactivity and functionality of bone graft materials, surpassing existing material limitations.

**Figure 1:**
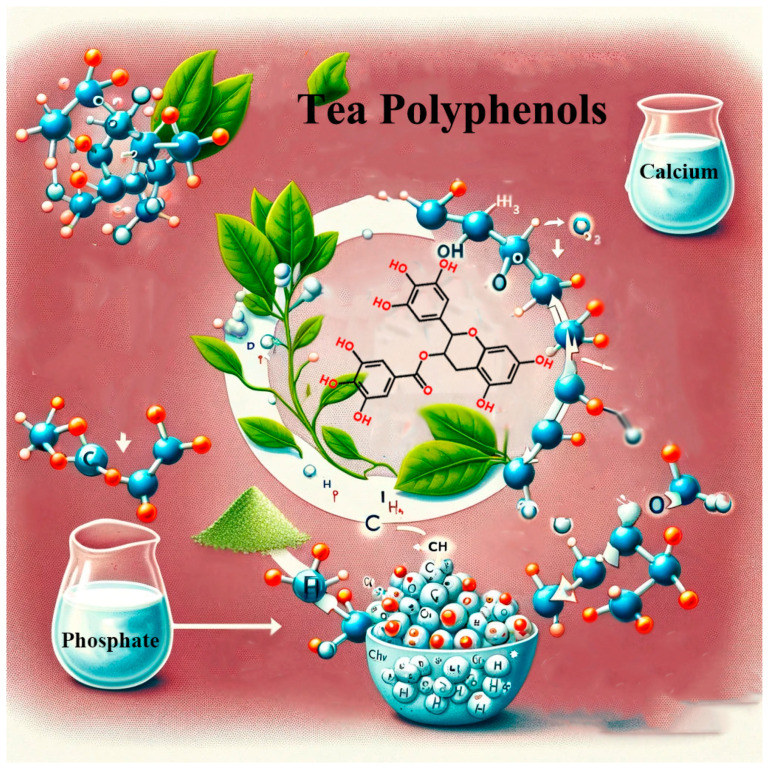
Schematic diagram of experimental process.

Previous investigations have underscored the pivotal role of the synthesis methodology in defining the characteristics of HAP. Among various methods, chemical precipitation stands out for its straightforward approach and economic viability, and is frequently employed in the production of HAP [30,31]. Nevertheless, mastering control over the nanoparticles’ size, shape, and crystalline structure during the synthesis process is still a complex undertaking. The aim of this study is to fabricate tea polyphenol (TP)-integrated HAP nanocomposites and to discern the effects of different synthesis parameters, such as the ambient temperature and the nature of the gas atmosphere, on their physicochemical attributes (Figure 1). Leveraging the chemical precipitation technique, this investigation delves into the synthesis of TP-enriched HAP and scrutinizes the implications of synthesis variables like temperature and gas milieu on the material’s qualities. The biological reactivity of HAP nanoparticles is heavily influenced by their crystallinity, morphology, and size [32,33]. Consequently, this study employs a suite of characterization tools—scanning electron microscopy (SEM), Fourier-transform infrared spectroscopy (FT-IR), and X-ray diffraction (XRD)—to meticulously assess the engineered nanomaterials. Through methodical adjustment of the synthesis conditions and thorough analysis of the materials produced, this research endeavors to shed light on the fine-tuning of TP-modified HAP’s properties, aiming to enhance its suitability for biomedical applications. Such an approach has the potential to revolutionize bone tissue engineering, offering a sophisticated solution that not only supports bone regeneration but also enhances the biological response, paving the way for advancements in bone repair surgeries and treatments.

## 2. Results and Discussions

### 2.1. SEM Analysis of Morphology

**Figure 2:**
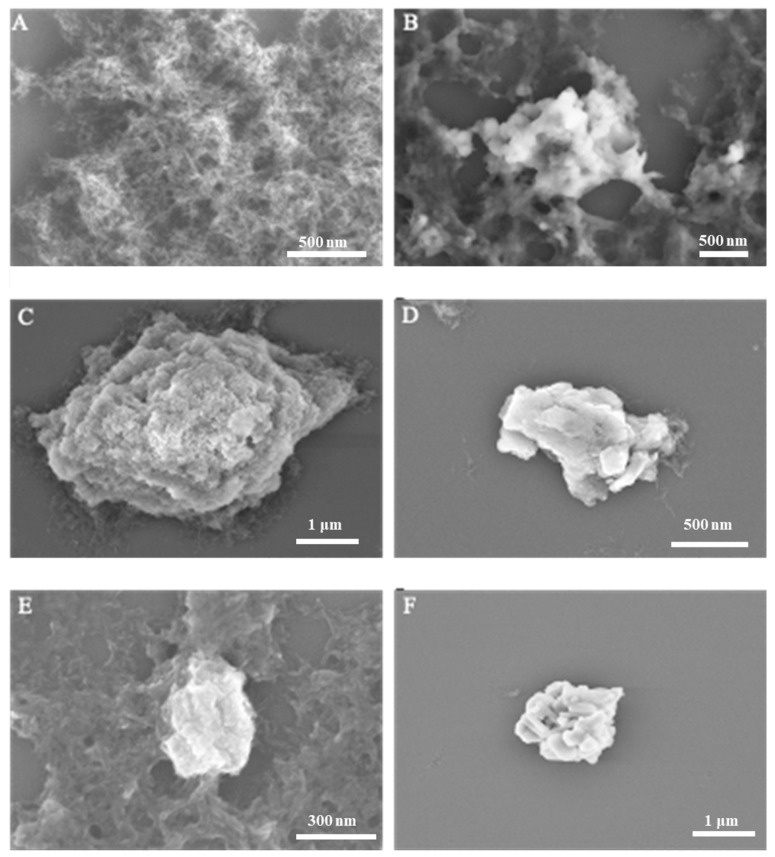
SEM images of the obtained samples. (**A**) Sample 1, pure HAP synthesized under 60 °C. (**B**) Sample 2, TP/HAP synthesized under 40 °C. (**C**,**D**) Sample 3, TP/HAP synthesized under 60 °C. (**E**) Sample 4, TP/HAP synthesized under 80 °C. (**F**) Sample 5, TP/HAP synthesized under 100 °C.

The morphological assessment of HAP synthesized without tea polyphenol (TP) intervention (Figure 2A) displayed a flocculent structure, diverging from the typically observed morphologies from hydrothermal and ammonium hydroxide diffusion methods. This divergence may be attributed to the synthesis being conducted at atmospheric pressure and the relatively short duration of the regulatory process. Figure 2B–F illustrate HAP that has been modified with TP at varying temperatures, predominantly featuring rod-like nanoparticles with increased aggregation density. The product obtained at 40 °C (Figure 2B) appears as irregular agglomerates with a loose and non-compact assembly. At 60 °C (Figure 2C,D), the HAP exhibits increased density with a potential irregular block-like stacking, forming flower-like patterns, although the compactness remains relatively low. Further temperature elevation to 80 °C and 100 °C (Figure 2E,F) does not significantly alter the morphology beyond a denser appearance, maintaining the irregular agglomerated form.

Comparing Figure 2A with Figure 2C,D, it is evident that TP regulation induces agglomeration in HAP, resulting in irregular aggregates. The observed temperature-dependent morphological changes suggest that TP not only affects the aggregation state of HAP but may also play a role in stabilizing certain morphological features at higher temperatures. These findings highlight the significance of TP as a regulatory agent in HAP synthesis, affecting the material’s structure and potentially its functional properties. Understanding the influence of TP on HAP morphology opens avenues for the development of tailored biomaterials with specific applications in mind, such as bone tissue scaffolding or drug delivery carriers.

### 2.2. XRD Crystallinity and Phase Composition

**Figure 3:**
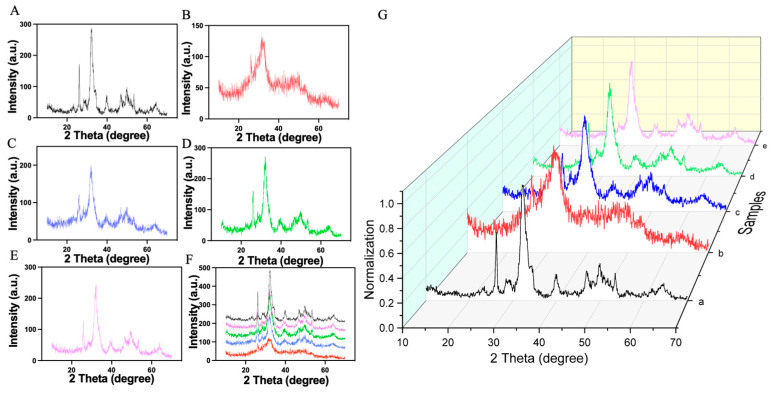
XRD spectra of samples. (**A**) Sample 1, pure HAP synthesized under 60 °C. (**B**) Sample 2, TP/HAP synthesized under 40 °C. (**C**) Sample 3, TP/HAP synthesized under 60 °C. (**D**) Sample 4, TP/HAP synthesized under 80 °C. (**E**) Sample 5, TP/HAP synthesized under 100 °C. (**F**) The combination of samples 1–5. (Sample 1/black curve, sample 2/red curve, sample 3/blue curve, sample 4/green curve, sample 5/pink curve). (**G**) Normalization of samples 1–5.

The XRD results for the synthesized samples are shown in Figure 3. To confirm the identity of the HAP produced, we compared the XRD pattern of pure HAP with the standard HAP. We observed minor peak shifts, yet the overall pattern closely matched the standard, suggesting the sample is predominantly HAP. The good overall agreement with the standard HAP pattern is evidenced by characteristic absorption peaks at 2θ values of 23.38°, 26.14°, 28.36°, 32.08°, 32.37°, 32.97°, 34.29°, 39.82°, 46.84°, 49.89°, and 53.38°, corresponding to the (111), (002), (102), (211), (112), (300), (202), (222), (213), and (004) crystal planes, respectively.

When the XRD patterns of all five samples were overlaid at the same scale (Figure 3G), all samples showed a high degree of overlap. This indicates a well-crystallized HAP structure, as evidenced by a prominent main peak at 32.08° and a notable side peak at 26.14°. However, sample 2 exhibited only a weak peak near 32°, indicative of poor crystallinity. We hypothesize that due to the addition of TP and the relatively low temperature of 40 °C, HA did not crystallize well, resulting in a sample with inferior crystallinity (Table 1). The findings underscore the impact of synthesis conditions, particularly temperature and duration, on the crystallinity of HAP. This crystalline quality is crucial for the potential application of HAP in biomedical fields, where precise material properties can significantly affect performance and integration into biological systems. The study demonstrates the importance of optimizing synthesis parameters to achieve high-quality HAP for advanced applications.

### 2.3. Chemical Structure of HAP/TP Nanoparticles

**Figure 4:**
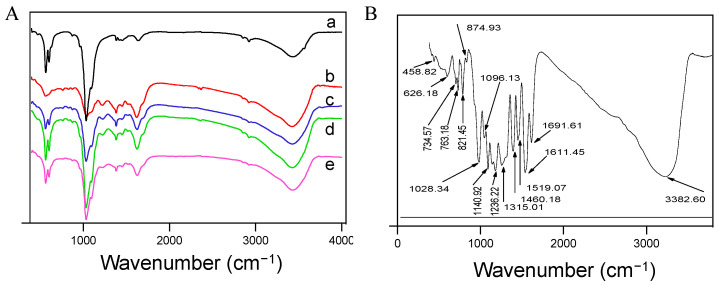
FTIR spectra of samples. (**A**) a, Sample 1, pure HA synthesized under 60 °C. b, Sample 2, TP/HA synthesized under 40 °C. c, Sample 3, TP/HA synthesized under 60 °C. d, Sample 4, TP/HA synthesized under 80 °C. e, Sample 5, TP/HA synthesized under 100 °C. (**B**) Pure TP.

FT-IR spectroscopy was employed to analyze the structural composition of five synthesized HAP samples (Figure 4). The spectrum labeled ‘a’, corresponding to pure HA, exhibited multiple absorption peaks. Notably, peaks at 3438 and 1638 cm^−1^ were assigned to O-H stretching and bending vibrations, indicative of adsorbed water, suggesting that the samples retained a small amount of moisture. Peaks at 868 and 1385 cm^−1^ aligned with characteristic vibrations of carbonate, implying the incorporation of CO_2_ into the system. This could be attributed to a lack of protective measures against CO_2_ ingress during the aging process, despite nitrogen protection during synthesis.

Characteristic HA absorption peaks were observed at 566, 603, and 1035 cm^−1^, associated with bending vibrations and asymmetric stretching of PO_4_^3−^. These findings are consistent with the XRD results, affirming the sample’s identity as HA. For the pure tea polyphenol (TP) spectrum (Figure 4B), a plethora of absorption peaks were present. Peaks at 3383, 1692, 1611, and 1315 cm^−1^ are related to various O-H vibrations from the adsorbed water which is physically bound to the surface of TP/HAP and the crystalline water which is located within the crystal lattice of TP/HAP, confirming the presence of moisture within the TP material. The range between 1450–1600 cm^−1^ represents characteristic vibrations of the phenolic ring structure. Peaks around 1028, 1096, 1141, and 1236 cm^−1^ could correspond to C-O stretching vibrations of phenols, and the latter to O-H in-plane bending. Peaks below 900 cm^−1^ suggest alterations in the aromatic ring, potentially due to oxidation processes that retain the aromatic framework but alter the substituents and reduce phenolic hydroxyl groups.

When TP was integrated into HA to form composites, the FT-IR spectra (Figure 4A (b–e)) showed changes similar to pure HA, with absorption peaks for water and CO_3_^2−^. Peaks around 565, 602, and 1030 cm^−1^ indicated HA’s characteristic vibrations, while peaks at 768, 822, 1524, and 1627 cm^−1^ were characteristic of TP, suggesting effective incorporation into the HA matrix.

Combining the XRD and FT-IR analyses, we conclude that this methodology yields HA with good crystallinity and effective TP integration. These results not only confirm the successful synthesis of TP-modified HA but also emphasize the potential of TP to modify the physical and chemical properties of HA, which could be critical for enhancing its application in biomedical fields.

### 2.4. TG Analysis for HAP/TP Content

**Figure 5:**
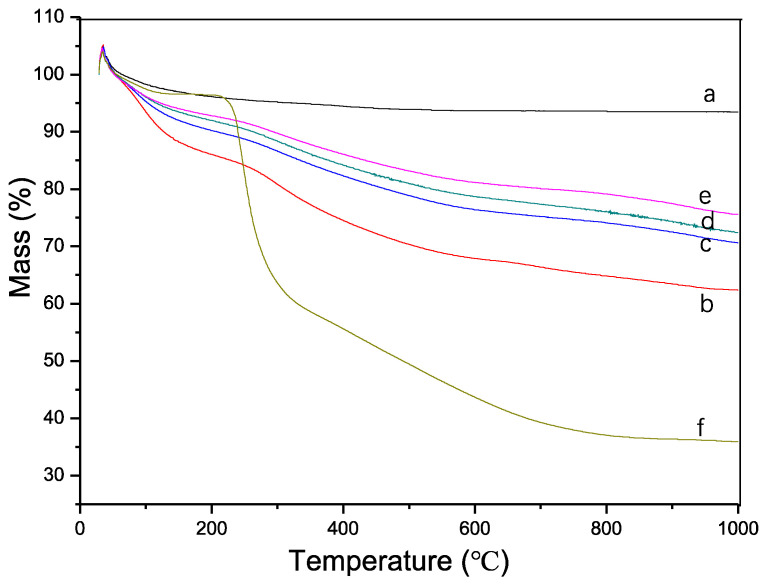
TG results of samples. a, Sample 1, pure HA synthesized under 60 °C. b, Sample 2, TP/HA synthesized under 40 °C. c, Sample 3, TP/HA synthesized under 60 °C. d, Sample 4, TP/HA synthesized under 80 °C. e, Sample 5, TP/HA synthesized under 100 °C. f, pure TP sample.

Thermogravimetric analysis (TGA) of the synthesized samples revealed an initial minor weight gain upon heating, which can be attributed to several factors. Firstly, for samples b-f, the reaction of tea polyphenol (TP) with atmospheric oxygen during heating could have resulted in oxygen incorporation, leading to a slight increase in mass (Figure 5). Secondly, the design of the TGA instrument, being of a vertical lift type, may have contributed to weight increase due to residual carbon falling onto the balance. Thirdly, an inaccurate instrument baseline may have affected the readings.

The TGA curve ‘a’ indicates that the synthesized HA demonstrates good thermal stability with a gradual and slight weight loss as the temperature increases, likely due to the evaporation of bound water. The weight stabilized at approximately 500 °C. Curve ‘f’ corresponds to the TGA curve of the pure TP material. TP is known to have a degree of thermal stability; however, it begins to thermally decompose between 200–600 °C. The significant weight loss commencing around 200 °C and continuing up to 800 °C suggests the decomposition of TP’s thermally unstable components, aligning with TP’s known properties after accounting for a lag effect.

From curves b-e, it is observed that the amount of TP integrated into the HA decreases with increasing synthesis temperature, consistent with the XRD results. Given the near-complete decomposition of TP’s active components at 800 °C, this temperature was selected to calculate the TP binding rate for each sample using the following formula. These results suggest that temperature is a critical factor in the synthesis process, affecting the integration of TP into HA. The decrease in TP content with increased temperature may reflect a higher rate of TP decomposition or a change in the interaction dynamics between TP and HA at elevated temperatures. Understanding these interactions is crucial for optimizing the synthesis process to enhance the material properties of HA for targeted biomedical applications.

The regulation of calcium phosphate by TP is inherently complex, and our experimental results did not yield the uniform particle distribution typically reported in the literature with grape seed polyphenol (GSP) regulation. The reasons for this discrepancy could be multifaceted, prompting further experimentation to investigate these causes. Additionally, the regulatory role of TP on calcium phosphate undergoes changes with varying temperatures. In alkaline conditions, TP binds to calcium phosphate through complexation, and temperature fluctuations can impact this binding process, leading to a range of differences in the reaction products. These differences are manifested as: A decrease in the binding rate of TP with an increase in temperature. Consequently, as the amount of TP integrated into the HA system decreases, the amorphous content theoretically should also reduce, resulting in increased crystallinity. This is consistent with the results obtained from the XRD analysis. Through the XRD and FT-IR analyses, we can confirm the successful synthesis of HA, with TP effectively incorporated into the HA matrix. From these results, we conclude that lower temperatures are more conducive to the integration of TP into the HA matrix, albeit resulting in HA with lower crystallinity. Conversely, at higher temperatures, the binding rate of TP to HA is reduced, but the crystallinity of the resulting HA is higher. These findings suggest that the synthesis temperature is a critical parameter for controlling the TP incorporation and crystallinity of HA. It emphasizes the necessity of temperature optimization in the synthesis process to achieve desired HA material properties for specific biomedical applications. Understanding the temperature-dependent behavior of TP in HA synthesis could facilitate the development of HA composites with tailored features for enhanced performance in their intended applications.

## 3. Materials and Methods

### 3.1. Chemical Reagents

Reagents used included tea polyphenol (TP, China Tea Institute, TP-98B, Hangzhou, China), anhydrous calcium chloride (analytical grade, Sigma-Aldrich, St. Louis, MO, USA), sodium phosphate dodecahydrate (analytical grade, Sigma-Aldrich), and ammonium hydroxide (analytical grade, 25%, Sigma-Aldrich). All solvents were prepared using deionized water with a resistivity greater than 18.3 MΩ·cm. The instruments utilized were a micropipette (Eppendorf, Hamburg, Germany), a high-speed tabletop centrifuge (H-1850, Xiangyi Centrifuge Instrument Co., Ltd., Changsha, China), a vortex mixer (IKA, Genius, Guangzhou, China), a constant-temperature magnetic stirrer (81-2 type, Sile Instrument Co., Ltd., Shanghai, China), a conductivity meter, an optical microscope, a freeze dryer, and various magnetic stir bars.

### 3.2. Synthesis Methods for TP/HAP Nanoparticles

HAP was synthesized via chemical deposition. Experiments were conducted at temperatures ranging from 40 °C to 100 °C in 20 °C increments to explore the regulatory effects of temperature on HAP, with parameters and sample numbers as per Table 2. For example, in Group 1, reagents were freshly prepared and used immediately, following these steps: Tea polyphenol solution (200 mL, 0.5%, *m*/*m*), anhydrous calcium chloride solution (0.1 M, 58.82 mL), and sodium phosphate dodecahydrate solution (0.1 M, 35.29 mL) were stirred in deionized water.

The reaction was conducted in a 500 mL three-neck flask. After filling the system with nitrogen through a condensation tube, deionized water (200 mL) was added, followed by 1.005 g of tea polyphenol, and stirring was initiated using a magnetic stirrer. An equal pressure funnel was then connected to introduce anhydrous calcium chloride solution slowly. Ammonium hydroxide was added to adjust the pH to 9. After stirring for half an hour to obtain a uniform chelate complex of calcium ions and polyphenols, sodium phosphate dodecahydrate solution was added dropwise at a rate of 1 mL/min. Ammonium hydroxide was used again to maintain the pH at 9 after the addition. The suspension was then heated to 40 °C and reacted for 2 h under nitrogen protection, with stirring speed maintained around 700 rpm. After the reaction, the solution was aged at room temperature for 72 h, then collected by centrifugation, and washed with deionized water until the conductivity dropped below 30 us/cm. A small part of the solution was dropped onto a silicon wafer, and the rest was freeze-dried for further characterization. For control, pure HAP was synthesized under the same conditions without tea polyphenol.

### 3.3. Scanning Electron Microscopy (SEM) Procedure

The study utilized a Hitachi S-4800 Scanning Electron Microscope to scrutinize the sample morphology meticulously. To begin the procedure, a minuscule quantity of each sample was meticulously diluted with deionized water inside an Eppendorf (EP) tube. This solution was then subjected to ultrasonication to ensure even dispersion of the sample particles. Following this, a droplet of the sample mixture was gently deposited onto a silicon wafer, serving as the substrate for SEM analysis. This wafer, with the sample droplet, was left undisturbed overnight in a controlled environment. This duration was crucial to ensure the complete and natural evaporation of water, leaving behind a dry sample perfectly suited for high-resolution SEM imaging.

### 3.4. X-ray Diffraction (XRD) Analysis

The crystalline structure and phase purity of the calcium phosphate in the samples were verified using X-ray diffraction, employing a DX-1000 X-ray diffractometer outfitted with CuKα radiation (wavelength λ = 1.5406 Å). The operational parameters for the XRD analyses were meticulously set to a voltage of 40 kV and a current of 25 mA. To prepare the samples for XRD, an adequate quantity of freeze-dried powder was first ground into a fine consistency using a mortar. This powder was then transferred into EP tubes, each meticulously labeled for systematic analysis. The XRD patterns were meticulously recorded over a range of 10° to 70° at a controlled scanning speed of 0.06°/s. The device settings, including divergent, scatter, and limiting slits, were adjusted to 1°, 1°, and 2 mm, respectively, with a monochromator configuration of Ts-Td. The crystalline structures present in the samples were identified by comparing the obtained XRD patterns with standard data for Hydroxyapatite (HA) from the International Centre for Diffraction Data (PDF#09-0432). All data analyses and crystalline structure interpretations were facilitated using the Jade 6.5 software, ensuring the high precision and reliability of the results.

### 3.5. Fourier-Transform Infrared Spectroscopy (FT-IR) Analysis

The characterization of the samples was further augmented using Fourier-transform infrared spectroscopy (FT-IR) employing a Perkin-Elmer Spectrum One B System. This analysis was not limited to the samples recovered from the XRD but also included pure Tricalcium Phosphate (TP) to serve as a reference. For this analysis, each sample was uniformly dispersed in potassium bromide at a precise ratio of 1:100. The mixture was then subjected to meticulous grinding under an infrared lamp to achieve a homogeneous blend. This blend was then compressed into pellets at room temperature, applying a pressure of approximately 40 MPa to ensure compact and uniform pellet formation. Prior to recording the spectra, a background test was meticulously conducted to eliminate any potential interference, ensuring that the FT-IR spectra obtained were solely representative of the sample’s inherent chemical structure.

### 3.6. Thermogravimetric Analysis (TGA)

The thermal stability and composition of the sample groups, alongside pure Tricalcium Phosphate (TP), were thoroughly investigated using thermogravimetric analysis (TGA) conducted with a NETZSCH STA 449 C apparatus. Initially, the samples and TP were finely ground to ensure uniform heat distribution during the analysis. Precisely measured quantities, ranging between 3–5 mg of each sample, were carefully placed into Al_2_O_3_ crucibles (DSC/TG pan). The TGA was performed in a meticulously controlled heating furnace, where the samples were gradually heated from a starting temperature of 25 °C to an upper limit of 1200 °C. This heating process was conducted at a constant rate of 15 °C/min, under an air atmosphere, spanning a total duration of 78 min. This gradual and controlled heating regimen ensured a detailed characterization of the sample’s thermal degradation profile and compositional changes.

## 4. Conclusions

In conclusion, this study presents a significant advancement in the field of biomaterials through the strategic synthesis of Hydroxyapatite (HAP) nanomaterials, utilizing Tricalcium Phosphate (TP) as an innovative morphological and compositional modifier via a chemical deposition method. This approach successfully yielded rod-like HAP crystals with remarkably high crystallinity, as evidenced by comprehensive thermogravimetric analysis, indicating the substantial incorporation of TP within the HAP structure and suggesting a successful hybridization. The study not only highlights the influence of the synthesis environment on material properties but also demonstrates that the chemical deposition method supersedes ammonia diffusion in achieving superior crystallinity of HAP. The dual role of TP, as both a morphological modifier and a potential regulatory agent, opens new horizons for biomedical applications, including anti-tumor, anti-infection, and bone repair functionalities. These findings establish a solid foundation for further exploration into the biomedical potential of HAP/TP nanorods, underscoring their significance in advancing biomedical engineering.

However, while the research marks a notable leap in nanomaterial synthesis, it also acknowledges certain limitations and areas for future exploration. The study does not delve deeply into the chemical reaction specifics of the synthesis process, and the intricate details of TP’s integration within the HAP structure require more profound investigation. The chemical interaction dynamics between TP and HAP, the detailed understanding of reaction pathways, intermediate phases, and conditions favoring the desired crystal structure and morphology are crucial areas that need further exploration. Additionally, the structural stability, long-term biocompatibility, and detailed biomedical functionalization of these hybrid nanorods in biological systems are paramount for harnessing their full potential. As such, future research should focus on a comprehensive understanding of these aspects, paving the way for the optimization and fine-tuning of these innovative nanomaterials for specific therapeutic and regenerative applications in the biomedical field.

## Figures and Tables

**Table 1:** The crystalline analysis of samples.

Sample	Intensity	BG	Height	Crystallite Size	Crystalline (%)
1	287	491	951	161	66.0
2	147	265	111	16	29.5
3	200	698	356	54	33.8
4	271	821	577	58	41.3
5	246	800	579	63	42.0

**Table 2:** Sample preparation conditions.

Samples	TP (g)	CaCl_2_ (0.1 M, mL)	Na_2_HPO_4_ (0.1 M, mL)	Temperature (°C)	H_2_O (mL)
1	0	58.82	35.29	60	200
2	1.005	58.82	35.29	40	200
3	1.005	58.82	35.29	60	200
4	1.005	58.82	35.29	80	200
5	1.005	58.82	35.29	100	200

## Data Availability

Data is contained in the paper.
